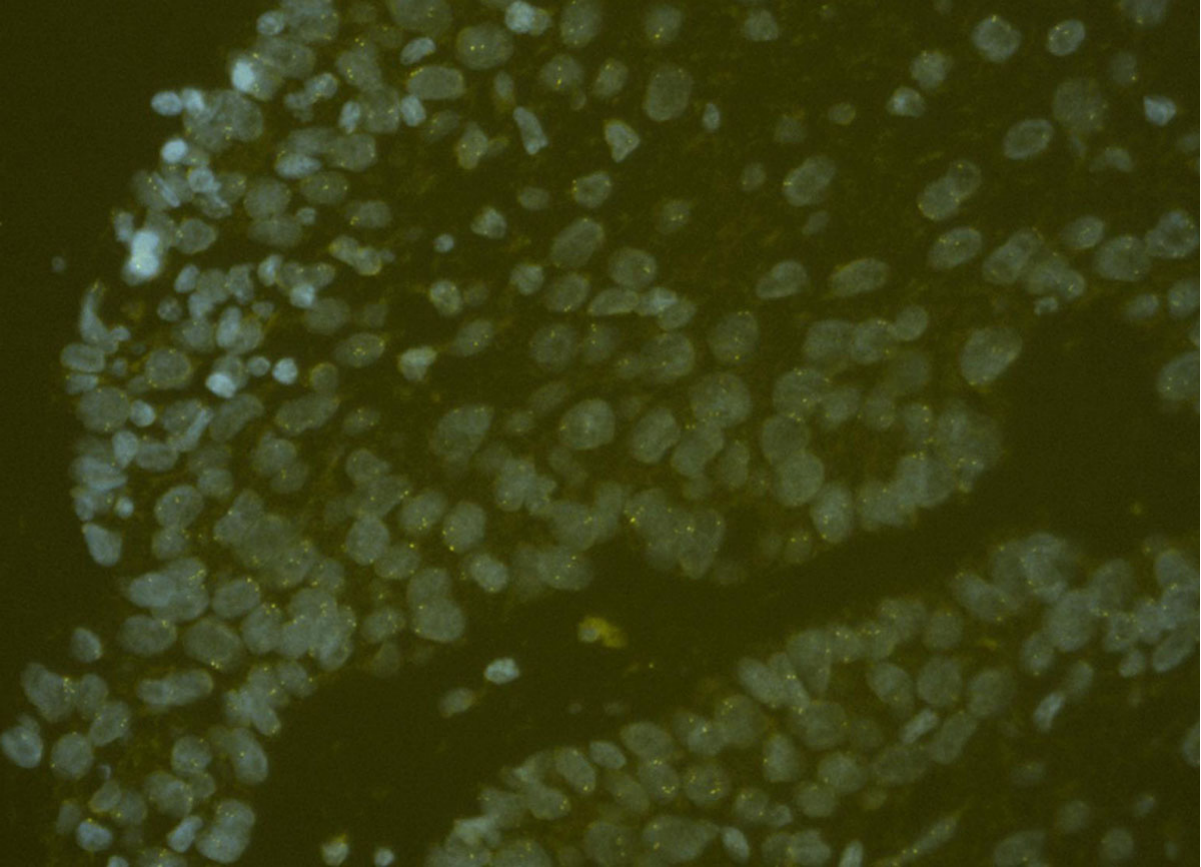# Evaluation of hTERC (3q26) gain by fluorescence in situ hybridization (FISH) to predict the behavior of oral epithelial precursor lesions

**DOI:** 10.1186/1878-5085-5-S1-A57

**Published:** 2014-02-11

**Authors:** Silvia Gabba, Elisa Giannini, Luisa Borghi, Giovanni Fellegara, Chiara Marcialis, Maria Grugni, Maria Pia Foschini, Elena Repetti, Tshering Dorji, Giorgio Vittadini, Vittorio Grazioli

**Affiliations:** 1 Laboratorio Analisi, Centro Diagnostico Italiano, Milano, Italy; 2 Anatomia patologica, Università degli Studi di Bologna, Italy; 3 Bracco Imaging, Centro Ricerche Bracco, San Donato Milanese, Italy

## 

Oral Squamous Cell Carcinoma (OSCC) is the most common tumor of the oral cavity. Approximately 500.000 new cases of OSCC are diagnosed worldwide each year with the incidence seemingly increasing. Chemo- and radiotherapy are potential curative procedures although destructive surgery is often necessary if early diagnosis is not achieved. Such procedures impact heavily on human and social costs. Infection of oral mucosa by oncogenic HPV is strongly associated with OSCC among subjects with established risk factors such as tobacco and alcohol abuse. The development of OSCC is a multistep process in which accumulated genetic and epigenetic events lead to cell cycle deregulation and chromosomal abnormalities. Gain of chromosome 3 (which contains human telomerase RNA gene, hTERC, located on chromosome 3q26) appears to be an important selective advantage in the progression of cervical intraepithelial neoplasia to invasive cancer [1] as well as in the progression to invasive head and neck carcinoma [2]. We utilized automated interphase FISH to evaluate the gain of chromosome 3 (3q26) and 7 (CEP) in oral epithelial precursor lesions in order to determine whether aneuploidy is already present and thus whether it is possible to anticipate diagnosis and clinical interventions. Retrospective in blind analysis on 40 oral samples with long-term follow up (ranging from 5 to 10 years) and with initial histological diagnosis of mild and moderate dysplasia, will be run. Preliminary results (8 samples) showed that tumor cells were already present only in the cases that progressed to cancer (4/8) [figure 1]; these preliminary findings confirm the usefulness of FISH as a reflex test in case of morphologically assessed pre-cancerous lesions, and highlight the potential value of this technique for early diagnosis and thus earlier treatment with less destructive therapeutic approaches.

**Figure 1:**